# In silico analysis of structural modifications in and around the integrin αIIb genu caused by *ITGA2B* variants in human platelets with emphasis on Glanzmann thrombasthenia

**DOI:** 10.1002/mgg3.365

**Published:** 2018-01-31

**Authors:** Xavier Pillois, Pierre Peters, Karin Segers, Alan T. Nurden

**Affiliations:** ^1^ Institut de Rhythmologie et de Modélisation Cardiaque, Plateforme Technologique d'Innovation Biomédicale Hôpital Xavier Arnozan Bordeaux France; ^2^ Université de Bordeaux INSERM U1034 Bordeaux France; ^3^ Laboratoire de Thrombose‐Hémostase Service d'Hématologie biologique et Immuno‐Hématologie CHU Sart Tilman Liège Belgium; ^4^ Service de Génetique CHU Sart Tilman Liège Belgium

**Keywords:** αIIb genu, genetic variants, Glanzmann thrombasthenia, in silico analysis, *ITGA2B*

## Abstract

**Background:**

Studies on the inherited bleeding disorder, Glanzmann thrombasthenia (GT), have helped define the role of the αIIbβ3 integrin in platelet aggregation. Stable bent αIIbβ3 undergoes conformation changes on activation allowing fibrinogen binding and its taking an extended form. The αIIb genu assures the fulcrum of the bent state. Our goal was to determine how structural changes induced by missense mutations in the αIIb genu define GT phenotype.

**Methods:**

Sanger sequencing of *ITGA2B* and *ITGB3* in the index case followed by in silico modeling of all known GT‐causing missense mutations extending from the lower part of the β‐propeller, and through the thigh and upper calf‐1 domains.

**Results:**

A homozygous c.1772A>C transversion in exon 18 of *ITGA2B* coding for a p.Asp591Ala substitution in an interconnecting loop of the lower thigh domain of αIIb in a patient with platelets lacking αIIbβ3 led us to extend our in silico modeling to all 16 published disease‐causing missense variants potentially affecting the αIIb genu. Modifications of structuring H‐bonding were the major cause in the thigh domain although one mutation gave mRNA decay. In contrast, short‐range changes induced in calf‐1 appeared minor suggesting long‐range effects. All result in severe to total loss of αIIbβ3 in platelets. The absence of mutations within a key Ca2+‐binding loop in the genu led us to scan public databases; three potential single allele variants giving major structural changes were identiffied suggesting that this key region is not protected from genetic variation.

**Conclusions:**

It appears that the αIIb genu is the object of stringent quality control to prevent platelets from circulating with activated and extended integrin.

## 1 INTRODUCTION

Integrins are cell membrane proteins composed of a series of α‐ and β‐subunits that noncovalently associate into 24 αβ heterodimers with divalent cation‐dependent adhesive functions (Horton et al., 2015; Hynes, 2002). αIIbβ3 mediates platelet aggregation by binding fibrinogen (Fg) or other adhesive proteins; in contrast, the closely related αvβ3 is more generally expressed and has wider functional diversity (Coller & Shattil, 2008; Ley, Rivera‐Nieves, Sandborn, & Shattil, 2016). αv and αIIb have 4 major extracellular domains (β‐propeller, thigh, calf‐1 and calf‐2) while β3 has 8 (βI, hybrid, PSI (plexin/semaphorin/integrin), 4 EGF (epidermal growth factor) domains and the β‐tail). The NH_2_‐terminal β‐propeller of αIIb (or αv) and the βI (sometimes called βA) domain of β3 associate to form a ligand‐binding globular headpiece; biochemical and biophysical studies all show that the heads are attached to legs (or stalks) with single pass transmembrane sequences and cytoplasmic tails distinctive of each subunit (Adair & Yeager, 2002; Carrell, Fitzgerald, Steiner, Erickson, & Phillips, 1985; Xiao, Takagi, Coller, Wang, & Springer, 2004; Xiong et al., 2001, 2009; Ye et al., 2010; Zhu et al., 2008).

Although different biophysical approaches have given varying interpretations of subunit domain arrangements, crystal structures show αvβ3 and αIIbβ3 extracellular domains with a bent conformation in their resting state with the closed head pointing downwards toward the membrane (Takagi, Petre, Walz, & Springer, 2002; Zhu et al., 2008). The major bend occurs at the αIIb genu situated between the thigh and calf‐1 domains and the “knee” of β3 where a link between I‐EGF‐1 and I‐EGF‐2 is at the epicenter of conformational change. Recent studies from Coller and his coworkers using purified αIIbβ3 in a nanodisc bilayer also suggest a bend involving αIIb calf‐1 and calf‐2 domains and an involvement of β3 I‐EGF‐2‐4 domains (Choi, Rice, Stokes, & Coller, 2013; Coller, 2015). Notwithstanding the model, αIIbβ3 structure is flexible and multiple conformational changes in response to activation lead to opening of the head and, for the majority of authors, a “switchblade” straightening of the extracellular domain (Choi et al., 2013; Coller, 2015; Li et al., 2017; Xiao et al., 2004; Zhang & Chen, 2012; Zhu, Zhu, & Springer, 2013; Zhu et al., 2008). This permits high affinity ligand binding and facilitates adhesive functions. While much is known of the structure of the αIIbβ3 headpiece, less is known about the molecular interactions at the fulcrum where the legs bend in the resting integrin (Blue et al., 2010; Smagghe, Huang, Ban, Baker, & Springer, 2010; Xiong et al., 2009).

Glanzmann thrombasthenia (GT, MIM #273800) is an autosomal recessive inherited platelet disorder characterized by excessive bleeding and quantitative or qualitative deficiencies of αIIbβ3 (George, Caen, & Nurden, 1990). As a result, platelets do not bind Fg and fail to form the bridges which crosslink them in an aggregate. Molecular screening of *ITGA2B* (MIM #607759) and *ITGB3* (MIM #173470), the genes that encode for αIIb and β3, has identiffied many genetic causes of GT (splice variants, insertions, deletions, nonsense, and missense mutations) (Buitrago et al., 2015; Nurden, Fiore, Nurden, & Pillois, 2011; Nurden & Pillois, 2017; Nurden et al., 2015). Mostly these cause a lack (type I GT) or much reduced αIIbβ3 expression (type II GT), although occasional qualitative variant forms have provided much information on how αIIbβ3 functions (reviewed in Coller & Shattil, 2008; Nurden et al., 2011). Significantly, a systematic analysis of *ITGA2B* and *ITGB3* variants identiffied in 16,108 individuals by new generation sequencing technologies has shown heterozygous expression of missense variants to be quite widespread in the normal population (Buitrago et al., 2015). Despite recent advances, questions of how critical is the genu for integrin expression and function remain largely unanswered. Our finding of a novel missense mutation in the genu region of αIIb in a Belgium patient with type I GT led us to review other, natural or engineered, published variants localized within amino acids (aa) 471‐769 incorporating the thigh and the upper calf‐1 domain of αIIb by using in silico modeling to look at their potential structural impact.

## 2 CASE HISTORY AND METHODS

### 2.1 Patients

#### 2.1.1 Index case

The index case is an adult Caucasian woman of Belgian nationality with type I GT whose platelets fail to aggregate with physiologic agonists (Wertz, Boveroux, Péters, Lenelle, & Franssen, 2011). She had a severe bleeding diathesis that included several episodes of gastric hemorrhage that twice resulted in hospital treatment. She developed antibodies to αIIbβ3 after platelet transfusions and was operated for spheno‐orbital meningioma under the cover of recombinant Factor VIIa. Some 8 days later she developed internal jugular venous thrombosis probably linked to catheter use (Wertz et al., 2011). Standard flow cytometry confirmed the lack of αIIbβ3 on her platelets but trace amounts of β3 suggested that αvβ3 was conserved. DNA was extracted with the QIamp DNA Blood Mini kit (Qiagen, Courtaboeuf, France) from EDTA‐anticoagulated whole blood. Sanger sequencing of exons, splice sites and untranslated regions of the *ITGA2B* and *ITGB3* genes was performed according to our published procedures (Nurden et al., 2015). This study was performed in accordance with the declaration of Helsinki after written informed consent from the patient, and under the approved ethical guidelines from the French National Institute for Medical Research (INSERM; approval RBM‐04‐14).

#### 2.1.2 Other cases

We performed a literature research for missense variants affecting amino acids 471‐769 extending from the base of the β‐propeller, through the thigh to the upper calf‐1 domain of human αIIb using PUBMED (https://www.ncbi.nlm.nih.gov/pubmed) (Keywords: Glanzmann thrombasthenia). Clinical and biological data were obtained retrospectively from the cited publications and are summarized and referenced in Table S1. For a wider view we also researched missense variants from the Human Genome Mutation Database, the 1000 Genomes project, the United Kingdom 10K Whole Exome Sequencing project, and the National Heart, Lung and Blood Institute Exome Sequencing Project as assembled by Buitrago et al. (2015) and updated the information within control populations by searching ExAC (http://www.exac.broadinstitute.org) and Ensembl genome browsers (http://www.ensembl.org; Herrero et al., 2016) according to Institutional Review board guidelines.

#### 2.1.3 Nucleotide and amino acid numbering

Human Genome Variation Society (HGVS) nomenclature for cDNA and protein is used throughout unless stated otherwise. For nucleotide numbering, the A nucleotide of the ATG start codon was designated +1 (cDNA *ITGA2B* Genbank accession number NM_000419.3). For amino acid numbering, +1 starts with the initiating Met with signal peptide included. This is often followed by that of the mature protein (in parentheses), this involves subtracting 31 amino acids from the HGVS numbering of αIIb and 26 for β3. Amino acid numbering for the mature protein was previously used in the crystal structure of αIIbβ3 and subsequent in silico modeling (see Xiao et al., 2004; Zhu et al., 2013). Alamut Visual software version 2.9 (Interactive Biosoftware, Rouen, France) was the primary source (1) for evaluation of nucleotide and amino acid conservation between species and within other integrin α subunits, (2) for estimating the physical‐chemical difference between amino acids (Grantham score) and (3) for the pathogenicity of the newly substituted amino acid by SIFT (version 6.2), PolyPhen and Mutation Taster (Nurden et al., 2015).

#### 2.1.4 In silico modeling

Protein models were constructed using the PyMol Molecular Graphics System, version 1.3, Schrödinger, LLC (http://www.pymol.org) and 3fcs or 1uv9 pdb files for crystal structures of αIIbβ3 as described by us (Nurden et al., 2011, 2015). Amino acid changes are visualized in the rotamer form showing side change orientations incorporated from the Dunbrack Backbone library with the maximum probability. Sequences composing the genu region are not continuous and are defined by color codes in our molecular models and framed in Figs S1 and S2 that show the conservation of amino acid residues between species and other integrin α‐subunits.

## 3 RESULTS

### 3.1 A novel mutation affecting the αIIb genu causing type I GT

**Figure 1:**
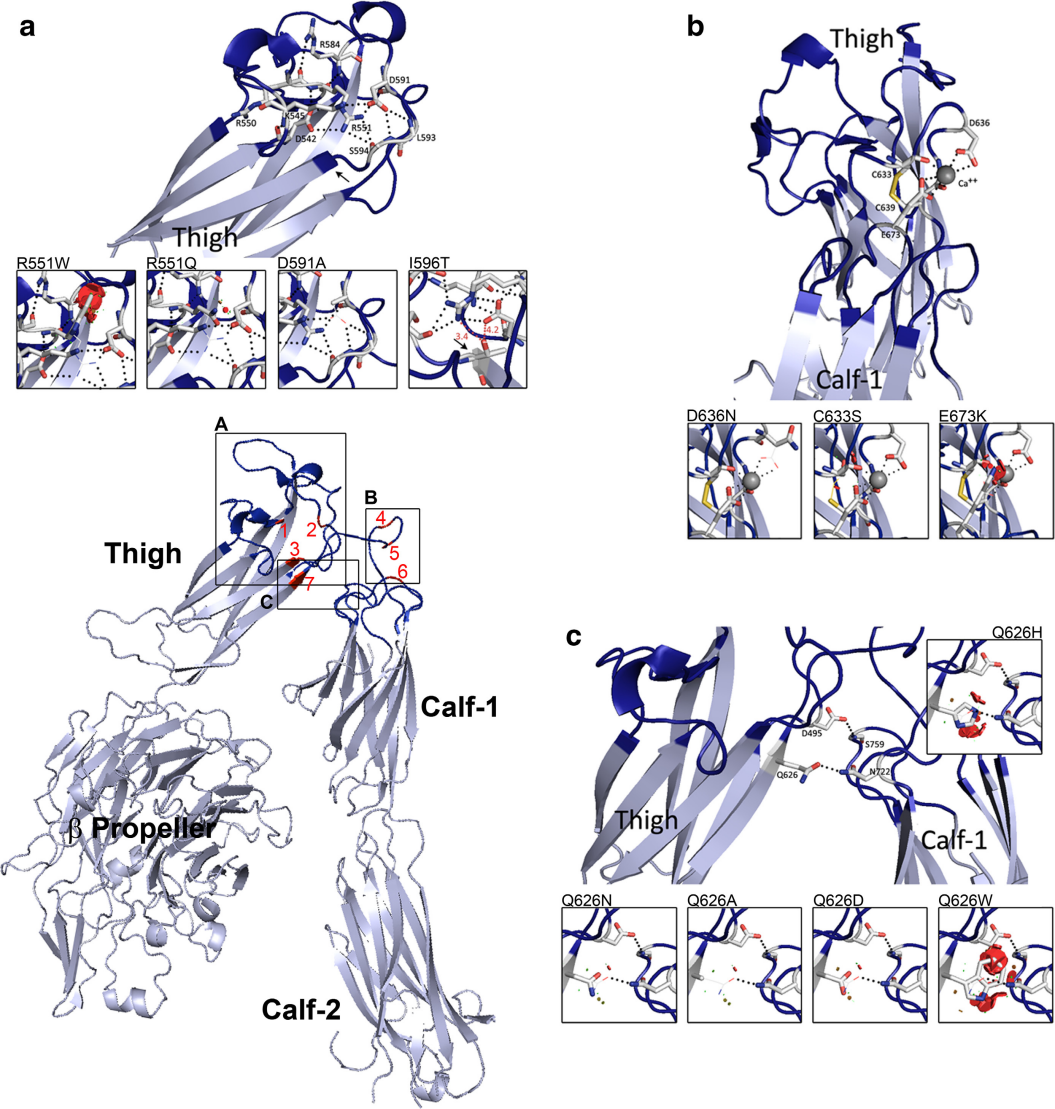
Ribbon diagrams highlighting selected missense mutations in and around the genu region of αIIb. The lower left panel shows a cartoon representation of the entire extracellular domain of αIIb with the genu in dark blue. Missense mutations most closely affecting this region are in red and numbered from 1 to 7. Framed are groups of missense variants within (a) connecting loops from the base of the thigh domain; (b) the unstructured linker ribbon between the thigh and the calf‐1 domains that contains a Ca2+ loop; and (c) the lower bend of the genu which contains a H‐bond clasp. In small windows are enlarged views of mutations illustrated as graphical sticks with superimposed the natural amino acid represented as lines. Graphical “bumps” (red discs) reveal steric encumbrance caused by the amino acid substitution. Amino acids engaged in H‐bonds (dotted lines) are represented as sticks with C atoms in white, N atoms in blue, O atoms in red and S atoms in orange. In (a) the black arrow indicates the position of Ile596; for Ile596Thr numbers in red are interatom distances in Angstroms, while a red dotted line represents a potential H‐bond introduced by the presence of Thr596. In (b) the Ca2+ is represented as a gray sphere and the dotted lines coordination links to nearby amino acids. Models were obtained using the PyMol Molecular Graphics System, version 1.3 and the 3fcs pdb file

Sanger sequencing of DNA from the index case, a Belgium type I GT patient, identiffied a homozygous c.1772A>C transversion (NC_000017.11:g.44379795T>G) affecting exon 18 of *ITGA2B*. It is a rare allele, featuring in one report on public databases, rs778608263 with a minor allele frequency (MAF) G = 0.000008354 (ExAC). This variant gives a p.Asp591Ala substitution (D560A for mature αIIb). Asp591 is highly conserved within vertebrates and is retained in all human integrin α‐subunits (Figs S1 and S2). The physical and chemical deviation between Asp and Ala is large (Grantham score: 126) and the mutation is described as “deleterious” by SIFT, “probably damaging” by PolyPhen and “disease causing” by Mutation Taster (Alamut Visual). αIIbD591 localizes to the lower part of the thigh directly affecting the intermediate or linker sequence composing the αIIb genu. As shown in Figure 1, the thigh domain is composed of seven antiparallel β strands forming a β barrel; connecting loops contain several polar amino acids whose side chains are involved in structuring H‐bonds. Among them, αIIbAsp591 shares H‐bonds with at least three neighboring amino acids (Arg551, Leu593 and Ser594). Its substitution by Ala results in the loss of these structuring H‐bonds (Figure 1a, inset). The fact that platelets from the patient lack αIIbβ3 indicates an early block in αIIbβ3 biosynthesis.

### 3.2 Distribution of known missense variants in and around the αIIb genu

**Figure 2:**
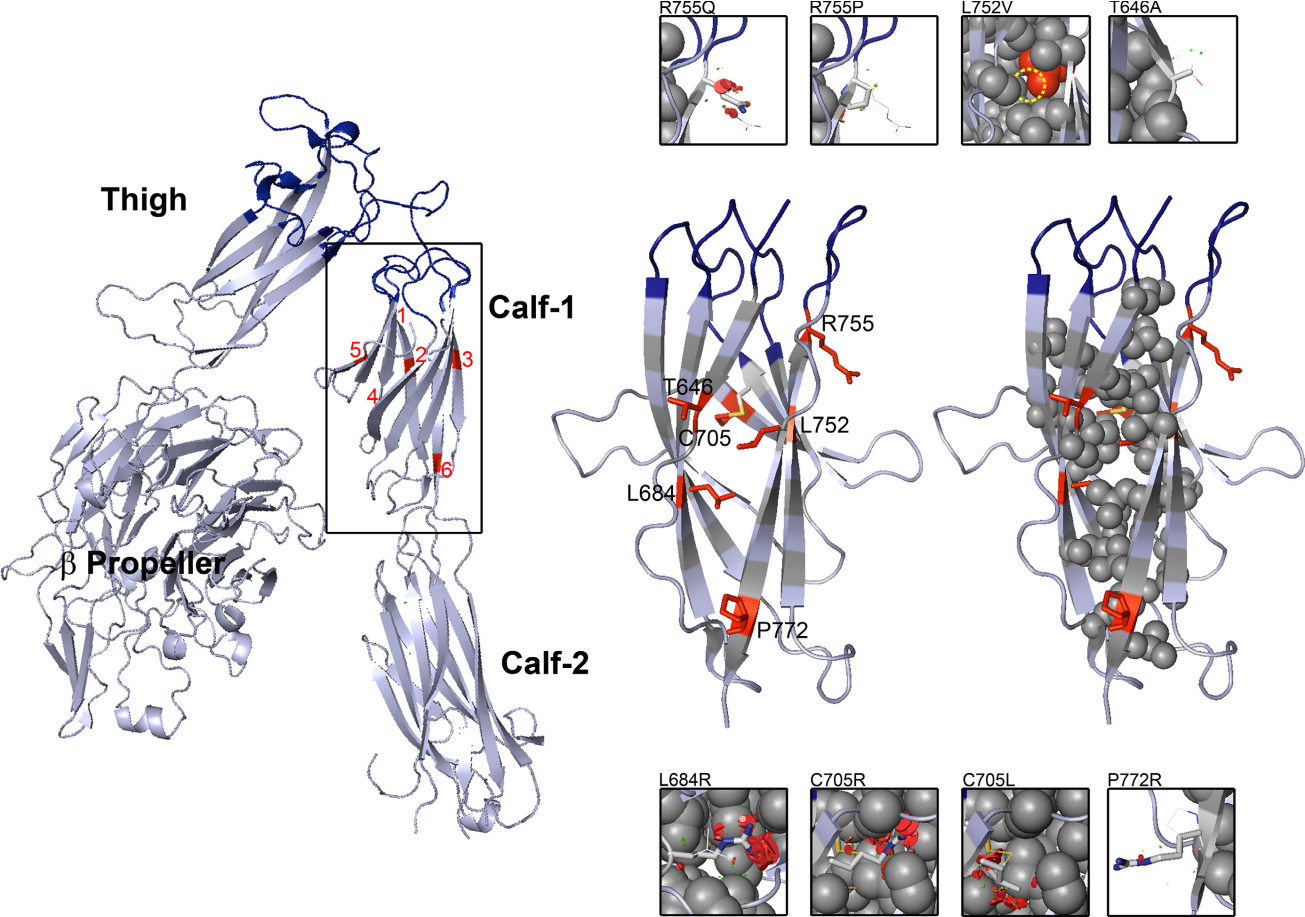
Cartoon representations of selected missense mutations within the αIIb calf‐1 domain. On the left hand side is a ribbon diagram depicting the extracellular domains of αIIb with the genu region colored in dark blue. Framed is the calf‐1 domain with missense mutations colored in red and numbered from 1 to 5. On the right hand side are selected representations of the calf‐1 domain with selected mutations as red sticks. The calf‐1 domain is structured as a β‐barrel composed of multiple anti‐parallel β‐strands. Amino acids whose side chain point to the core of the barrel are colored in gray and represented as spheres in the far right representations. In the small windows are enlarged views with each mutation illustrated as graphical sticks with superimposed, the natural amino acid represented as lines. C atoms are colored in white, N atoms in blue, O atoms in red and S atoms in orange. Graphical “bumps” (red discs) reveal steric encumbrance caused by the amino acid substitution. Models were obtained using the PyMol Molecular Graphics System, version 1.3 and the 3fcs and 1uv9 pdb files

A comprehensive search of the literature revealed 15 missense variants causative of GT within the aa471‐769 sequence that begins with the C‐terminal strands of the β‐propeller, extends through the thigh domain and terminates with the upper domain of calf‐1 (Table S1) (Figures 1 and 2). All were associated with type I or type II GT and absent or much‐reduced levels of αIIbβ3 in their platelets. Bleeding severity differed widely between these patients and independent of the site of the amino acid substitution. Two additional low frequency human platelet alloantigen (HPA) systems, Ser503Asn (HPA‐24b) in the thigh domain and Thr650Met (HPA‐20b) in calf‐1 fail to affect αIIbβ3 expression and are associated with a post‐transfusion immune response but not lack of function (Jallu, Dusseaux, & Kaplan, 2011; Peterson et al., 2010). Interestingly, of the 15 GT‐causing mutations only Ile596Thr featured in the list released by Buitrago et al. (2015) who analyzed nonsynonymous variants affecting *ITGA2B* and *ITGB3* identiffied by whole exome and whole genome human sequencing in over 16,000 normal individuals in the ThromboGenomics project. Nevertheless 16 (thigh) and 23 (calf‐1) nonsynonymous missense variants of unknown pathogenicity were present. A current assessment of the aa471‐769 sequence in the ExAC database (*ITGA2B*, 21/08/2017) revealed 97 missense variants. This increased to 134 when Ensembl was consulted; a list that now includes presumed heterozygous expression of eight of the 15 variants causative of GT when homozygous or part of compound heterozygosity. Clearly, the genu and surrounding regions of αIIb are undergoing constant evolutionary genetic change.

### 3.3 Structural consequences of missense variants within the thigh and its connecting loops

To better understand the pathological influence of the Asp591Ala variant we first performed in silico modeling of domains close to and including the upper connecting loops of the genu.

- *C‐terminal domain of the* β*‐propeller*. The only known disease‐causing β‐propeller mutation in our selected aa471‐769 sequence is a homozygous Ala477Pro (A446P) substitution in a Chinese boy with type II GT (Fu, Yuan, Chen, Xia, & Fu, 2005). Ala477 lies in the middle of the 3rd β‐strand of the seventh blade of the β‐propeller close to the link to the thigh domain (Fig. S3). This blade is clearly critical as it closes the circular propeller by interacting with blade 1; introduction of a Pro with its tendency to form a β‐turn into the center of a β‐sheet creates a tension that weakens H‐bonds that link the third and fourth β‐strands of blade 7 together. Site‐directed mutagenesis in CHO cells by Fu et al. (2005) showed that surface expression of αIIbPro477β3 was reduced to 14% of wild‐type levels. Western blotting showed pro‐αIIb to be synthesized and immunofluorescence labeling revealed that the mutated pro‐αIIbβ3 was retained in the ER. Interestingly, a near‐neighbor homozygous Ile458Met mutation in an Indian boy gave type I GT (Vijapurkar, Ghosh, & Shetty, 2009).
- *Disease‐causing thigh and linker‐domain mutations*. One of the H‐bonds of Asp591 (mutated in the index case above) is with Arg551 of αIIb (Figure 1a); Arg551 (R520) is a mutation hotspot for GT with homozygous substitution by Trp in two patients (cases 4 and 5) originating from Southern India (Nelson et al., 2006) or by Gln in a young boy from India (case 6) and five patients (cases 7–11) from three unrelated families in Pakistan (Haghighi et al., 2016; Viyapurkar et al., 2009) (Table S1). Both substitutions give type I GT and substantial bleeding. Arg551 is highly conserved both between species and within human integrin α‐subunits (Figs S1 and S2) and like Asp591 it localizes to a connecting loop of the lower region of the thigh domain. In fact, both substitutions of Arg551 lead to the loss of multiple structuring H‐bonds, with the larger Trp additionally introducing steric encumbrance (Figure 1a insets).
- Close to Asp591, Ile596Thr (I565T) has been the object of several reports mostly from Europe, giving rise to type I GT with moderate to severe bleeding (Table S1). Two cases have homozygous expression; in the other three, Ile596Thr is part of compound heterozygosity paired with IVS29(+2)T>C, c.3091delC or a well‐characterized Glu355Lys (E324K) missense mutation (French & Coller, 1997; Jallu et al., 2010; Ruan et al., 1998; Sandrock et al., 2012; Sandrock‐Lang et al., 2015). Thus Ile596 (localized on Figure 1a) is a likely mutational hot spot. Ile596 is relatively well conserved both between species and within human α‐subunits; when nonconserved the replacement amino acids have similar biochemical properties and steric encumbrance (Figs S1 and S2). The physical and chemical deviations between the amphipathic C5 Ile and the polar uncharged C4 Thr are modest (Grantham score: 89) and at first sight, in silico modeling suggests the impact of the Thr substitution to be minor. However, Thr introduces an ‐OH alcoholic function close (3.4Å) to Arg551, this competes for H‐bonds with αIIb residues Asp542, Ser594 and Asp591 changing interatom distances and creating an additional H‐bond within connecting loops extending from the lower region of the thigh (Figure 1a).
  A homozygous Pro507Arg (P476R) thigh domain substitution was detected in a patient from India (Peretz et al., 2006). Pro507 is poorly conserved (Figs S1 and S2) but the physical and chemical deviation between Pro and Arg is important (Grantham score: 103). Predicted to be deleterious by SIFT and possibly damaging by PolyPhen it is defined as a polymorphism by Mutation Taster confirming the unpredictability of these programs as highlighted elsewhere (Buitrago et al., 2015). As in silico modeling performed by the authors localized Pro507 to the tip of a loop and partially buried within the subunit, we have concentrated our modeling in this zone to Ile518Asp (I487D) and Ala581Asp (A550D). Ile518Asp (case 3, Table S1) is part of compound heterozygosity with a c.1946+3G>T splice site variant in a young American male with type I GT and severe bleeding (Nurden et al., 2015). It is predicted to be possibly damaging or deleterious. Ala581Asp (case 12, Table S1) also had heterozygous expression being the only mutation found in an Italian type I GT patient, suggesting a second nonidentiffied defect (D'Andrea et al., 2002). Like Pro507Arg, Ile518 and Ala581 locate to the thigh β‐barrel. Composed of seven β‐strands; the β‐barrel is largely made up of hydrophobic amino acids whose side chains point to the core of the structure whereas side chains of polar or charged amino acids point to the outside. Ile518 and Ala581 are hydrophobic and the introduction of a highly charged Asp in both cases introduces deep instability (Fig. S4) with a block in αIIbβ3 biosynthesis explaining its absence from the platelet surface and type I GT.

- *Ca2+‐binding loop in the genu*. A clearly defined structure of the αIIb genu is a Ca2+ loop with two Cys (Cys633 and Cys639) involved in a disulfide bridge and four amino acids engaging in Ca2+ coordination (Cys633, Asp636, Val638, and Glu673) (Figure 1b). At the present time, none of these amino acids are mutated in a GT patient. However, on performing a database search we found missense variants for 3 of them on the Ensembl database: Cys633Ser (rs1126555; no frequency data), Asp636Asn (rs749873100; MAF 0.00004) and Glu673Lys (rs368974006; MAF 0.00002/8). Although no biological or clinical information associated with these mutations is available, at least Cys633Ser is stated as being probably damaging by Polyphen, and our in silico modeling shows that Cys633Ser and Glu673Lys have a deep impact on αIIb structure (Figure 1b): the first by disrupting the disulfide bridge, and the second by affecting Ca2+‐binding. Thus structure‐modifying mutations directly affecting the αIIb genu are present already in the normal population showing that this region too is the object of evolutionary change.
- *Nonsynonymous variants in the thigh domain associated with mRNA loss*. Homozygous Gln626His (Q595H) giving type I GT was independently detected in four European studies suggesting a possible founder effect (Jallu et al., 2010; Nurden et al., 2015; Pillitteri, Pilgrimm, & Kirchmaier, 2010; Sandrock et al., 2012). In fact, the c.1878G>C transversion responsible for Gln626His primarily effects splicing with exon skipping leading to mRNA decay (Jallu et al., 2010; Nurden et al., 2015). In fact, when αIIbHis626β3 was co‐expressed with β3 in COS‐7 cells, surface expression of αIIbβ3 was normal (Jallu et al., 2010). In a parallel study, Kamata et al. (2010) engineered Gln626 substitution by Asn, Ala, Asp, and Trp among others and found that none of them prevented expression of the recombinant integrin in CHO cells; furthermore the mutated αIIbβ3 remained nonactivated and therefore bent. Because of the strategic position of Gln626 we performed in silico modeling to examine short‐range changes introduced by these engineered variants on integrin structure. Figure 1c shows how Gln626 forms a H‐bond with Asn722 in calf‐1 and together with near‐neighbor Asp495 that forms a H‐bond with Ser759 in calf‐1 constitutes a H‐bond clasp. His626 and Asn626 introduce modest structural changes and have little effect on H‐bonding (Figure 1c). While substitution of Gln626 with the smaller Ala, the oppositely charged Asp, or the bulky Trp cause loss or weakening of at least one of the H‐bonds, sufficient interaction is retained so that the clasp is not broken. Our in silico modeling therefore helps explain the retention of recombinant αIIbβ3 expression after transfection in heterologous cells.

### 3.4 Repertoire of variants in the calf‐1 domain

Calf‐1 has a β‐structure with nine β‐strands connected to the NH_2_‐terminus of the thigh domain through interconnecting loops. Our literature research identiffied nine disease‐causing missense variants within the genu‐proximal part of calf‐1 (Table S1). D'Andrea et al. (2002) reported Arg755Pro (R724P) coexpressed with Leu752Val (L321V) in a patient with type I GT; intriguingly both variants had homozygous expression suggesting a rare haplotype. Subsequently, homozygous Arg755Pro alone was found in a young Indian woman with mild bleeding and type I GT (Vijapurkar et al., 2009). While Arg755 is moderately conserved within species or human α‐subunits, Leu752Val is only weakly conserved suggesting a low importance for integrin biosynthesis and/or function (Figs S1 and S2). We identiffied heterozygous Arg755Gln in two siblings from the USA with type I GT and severe bleeding but very unusually both siblings also expressed two potentially heterozygous disease‐causing *ITGB3* mutations so the role of Arg755Gln in the GT phenotype is difficult to predict (cases 35a,b in Nurden et al., 2015). This is even more so as αIIbArg755Pro, Arg755Gln, and Leu752Val are all reported as tolerated by SIFT, probably or possibly damaging by PolyPhen and disease causing by Mutation Taster, again underlining the variability in the prediction algorithms. The effects of Arg775Pro, Arg755Gln, and Leu752Val on the structure of calf‐1 are first compared in Figure 2. Arg755 localizes to the upper end of a β‐strand with its side chain aligning toward the exterior limiting the short range structural effect within αIIb of its substitution by Gln or Pro. Significantly, long‐range effects were recently identiffied for these substitutions from molecular dynamics simulations (Goguet, Narwani, Petermann, Jallu, & De Brevern, 2017). The jury is out on whether the relatively inert Leu752Val contributes to the type I GT phenotype. At the interface between calf‐1 and calf‐2 Pro772Arg (and erroneously stated to be within calf‐2 in the original report) was not considered further by us.

Other missense mutations affecting calf‐1 concern residues that locate to β‐strands central to the β‐barrel (Figure 2) but few studies have investigated how they influence αIIb structure. Thr646Ala (T615A) was detected in a Turkish patient with type I GT (Tokgoz, Torun Ozkan, Caliskan, & Akar, 2015). Thr646 points to the exterior and its replacement by the smaller Ala on αIIbβ3 structure is difficult to predict. Leu684Arg (L653R) was found in a type I patient from Italy but with heterozygous expression, a presumed mutation affecting the second allele remains to be identiffied (Pillitteri et al., 2010). Leu684Arg concerns the hydrophobic core of the β‐barrel replacing a hydrophobic amino acid with a larger polar residue introducing steric encumbrance (red bumps on Figure 2), a change having a highly destabilizing effect. Of much interest is Cys705Arg (C674R) first detected as part of compound heterozygosity in a Spanish patient with type II GT (Gonzalez‐Manchon et al., 1999). The second allele for this patient had a splice site mutation, IVS5(+2)C>A that led to insertion of intron 5, mRNA instability, and a loss of αIIb. As is shown on Figure 2, substitution of Cys705 results in loss of the αIIbCys705‐Cys718 disulfide bridge; this renders the β‐barrel unstable promoting expression of the hydrophobic core at the exterior. The same variant was subsequently detected in three Italian families with homozygous expression in two of them and as part of nonidentiffied compound heterozygosity in a third (D'Andrea et al., 2002). Mitchell et al. (2003) independently identiffied homozygous Cys705Arg in another Italian man with type II GT. The situation in the latter family was complicated as αIIbIle405Thr formed part of compound heterozygous expression in other affected members (Mitchell et al., 2003). Heterozygous Cys705Arg has since featured in two later reports from France where it combined with intronic splice defects on the second allele that cause loss of expression (Jallu et al., 2010; Nurden et al., 2015). The location of many patients with Cys705Arg in Europe strongly suggests a founder defect. Finally, heterozygous expression of Cys705Leu (C674L) in a German patient with <5% αIIbβ3 has similar effects although for this patient a second allele mutation remains to be found (Santoro et al., 2010) (Figure 2). Interestingly, when αIIb705Arg was transfected with wild‐type β3 in CHO cells formation of the pro‐αIIbβ3 complex still occurred (Gonzalez‐Manchon et al., 1999). Although maturation and surface expression of the mutated complex was greatly disrupted there was sufficient expression to account for the type II phenotype.

## 4 DISCUSSION

Despite occurring in an a‐priori critical region for changing between resting and activated states, we show that disease‐causing variants within or near the αIIb genu are primarily associated with defects of αIIbβ3 maturation and a loss of surface expression. The genu links the calf‐1 and thigh domains (see Introduction) of αIIb whose key features suggested from the crystal structures of αvβ3 and αIIbβ3 include (1) multiple β‐sandwich folds, (2) intermediate loops extending from the base of the thigh to the top of calf‐1 and (3) an α‐β interface between the thigh and EGF‐domains (Xiong et al., 2001, 2009; Zhu et al., 2008). A well‐coordinated Ca2+ lies in an extended loop of the genu close to calf‐1 and may help neutralize opposing acidic patches at the α‐β subunit interface. Our finding of a novel αIIb substitution (Asp591Ala) in a Belgian patient with type I GT that concerned an amino acid localized within a connecting loop of the lower region of the thigh domain and influencing genu structure by participating in a highly structured network of H‐bonds highlights the potential structural importance of this region. This led us to review the effects of other natural amino acid substitutions within an arbitrarily chosen amino acid 471‐769 sequence extending down from the base of the β‐propeller through the thigh and the upper region of calf‐1. Significantly, H‐bond loss was a frequent finding with the disease‐causing mutations.

All 15 GT‐causing missense variants previously identiffied within the amino acid 471‐769 sequence resulted in loss or severely reduced αIIbβ3 expression in platelets (Table S1); a result confirmed in certain studies after expression of recombinant mutated integrin in heterologous cells (Fu et al., 2005; Gonzalez‐Manchon et al., 1999). Such results mimic those obtained for a much larger number of missense mutations detected in the β‐propeller head of αIIb (Nelson et al., 2005, 2006; for a more recent review see Nurden et al., 2011), but contrasts with the β1(or βA) extracellular domain of β3 where classic Asp145Asn/Tyr (D119N/Y), Arg240Gln/Trp (R214Q/W) substitutions allow αIIbβ3 expression but prevent formation of a functional ligand‐binding pocket (reviewed in Coller & Shattil, 2008; Nurden et al., 2011). No Fg‐binding domains have been located to the αIIb genu or near‐neighbor domains, although heparin and heparin sulfate may bind to integrins by way of Lys or Arg residues in this region offering a potential for modifying integrin function (Ballut, Sapay, Chautard, Imberty, & Ricard‐Blum, 2013; Yagi et al., 2012). Interacting platelets are sources of reactive oxygen species (ROS) able to modulate αIIbβ3 function by oxidizing cysteines as part of cellular redox regulation (Essex, 2009). Studies on α7β1 suggest that a primary site for ROS are Cys residues in the genu a finding that also has functional implications (de Rezende et al., 2012). Cytoplasmic domain missense mutations of αIIb (e.g. Arg1026Gln or Trp; R995Q/W) or β3 (e.g. Leu744Pro or Ser778Pro (L718P or S752P) can have long‐range effects on the extracellular domains either by abrogating “inside‐out” signaling pathways dependent on talin and kindlin‐3 binding to the β3 cytoplasmic tail or conversely lead to partially activated integrin and defects of platelet production (reviewed by Nurden et al., 2011; Nurden & Pillois, 2017). No platelet size variations were seen in our selected GT cohort.

In silico modeling of the αIIb Asp591Ala mutation in the index case showed a loss of close‐range H‐bonds including Arg551, itself a mutation hotspot for GT being substituted either by Trp or by Gln in several unrelated type I patients (Table S1). Asp591 and Arg551 localize to connecting loops between β‐sheets forming the β‐barrel structure of the thigh domain, proximal to the genu. Steric interference (particularly for Trp551) and a reduced positive charge weakened structured H‐bonds involving side chains of the mutated amino acids. H‐bond modifications were also induced by Ile596Thr another common mutation giving type I GT. Here the introduction of Thr influenced neighboring Ser594 as well as Arg551. In a parallel with the thigh, connecting loops of the upper region of calf‐1 also extend from β‐strands composing a β‐barrel; but here it is mostly the main body of the constitutive amino acids that engage in H‐bonds. This gives a tighter structure, less sensitive to amino acid substitutions, and with a greater potential importance for steric impact. Arg755Pro or Arg755Gln are well‐studied examples of calf‐1 missense substitutions (Table S1). Although substitution of αIIbArg755 by a Pro, an amino acid with a tendency to form a β turn may directly introduce destabilization, the fact that it points to the exterior of the β‐barrel makes this less likely. Immediately prior to submission of our manuscript, Goguet et al. (2017) published in silico analysis of variants within the calf‐1 domain using a dynamic approach. While confirming that the rigid nature of the calf‐1 backbone restricted short‐range conformational change for substitutions of Arg755 they additionally showed how long independent molecular dynamic simulations revealed distant allosteric changes that may contribute to the pathogenicity of calf‐1 substitutions and show that short‐range and long‐range changes both contribute to pathogenicity in GT.

The disease‐causing effect of αIIbGln626His has a different explanation. Located immediately behind the genu, it is the cause of type I GT in several unrelated families in Europe with a c.1878G>C transversion affecting splicing with loss of exon 28 resulting in truncated mRNA undergoing rapid decay (Jallu et al., 2010; Nurden et al., 2015). The fact that expression of one natural and four engineered αIIbGln626 variants with β3 in CHO cells had little effect on integrin surface density (Jallu et al., 2010; Kamata et al., 2010) led us to perform in silico modeling. Although Gln626Asn and Gln626Asp have opposing charge effects the substituted amino acids retain their ability to form H‐bonds with their counterpart Asn722 while that between Asp495 and Ser759 is also retained, findings that may explain the absence of a notable effect on expression after mutagenesis. Even in the case of substitution of Gln626 with the smaller Ala and specific loss of the H‐bond with Asn722, no impact on integrin expression or its activation state was reported (Kamata et al., 2010). Even more intriguingly, introduction of the bulky Trp that disrupts the H‐bond not only with Asn722 but which pushes out the nearby H‐bond between αIIbAsp495 and Ser759 failed to prevent integrin expression. Therefore in each case sufficient structural integrity was retained to over‐ride the quality control systems of the CHO cells during integrin maturation. Interestingly, studies by Kamata et al. (2010) also showed the recombinant integrins to be retained in a non‐activated (bent) form, except when substitution by Asn created a N‐glycosylation site, the bulky N‐glycan now being sufficient to cause swing‐out of the hybrid domain, integrin extension, and partial activation.

A striking feature of the α‐subunit genu is a short, disulfide‐bonded loop first identiffied between residues 746 and 752 in αL and corresponding to residues 633 and 639 in αIIb (see Figure 1b) and a Ca2+‐binding site that is reportedly occupied in both the bent and extended forms (Xie et al., 2004; Zhu et al., 2008). Coordination to genu Ca2+ in αIIb is provided by the backbone carbonyl of Cys633 and Val638, the side‐chain of Asp636 and the side‐chain of Glu673 in calf‐1 (Zhu et al., 2008). Knowledge of this region was advanced by measuring binding of two activation‐dependent antibodies before and after activation of αLβ2 (LFA‐1) (Xie et al., 2004). In the bent conformation the αL‐subunit calf domain and the nearby I‐EGF domains in the β leg shield key innermost thigh residues for antibody binding that become exposed on extension. On activation, the αL genu locks into an extended, rigid, conformation with Ca2+ also favoring antibody binding (Xie et al., 2004). The structural rearrangement at the genu was concluded to occur between the thigh domain and the genu rather than between the genu and the calf‐1 domain as the Ca2+‐coordinating residue in calf‐1 is maintained in the extended conformation. Within the bent αv conformation the Ca2+‐coordinating residue Glu636 (corresponding to αL calf‐1 residue Glu787) is at the center of a H‐bonding network linking the genu to the calf‐1 domain. Thus αv Glu636 coordinates both the Ca2+ and the genu residue 603 backbone with its side chain and also forms the backbone‐backbone H bond to residue 603. No such interactions exist between the genu and the thigh. No disease‐causing mutation of amino acids engaged in Ca2+ coordination in αIIb (Cys633, Asp636, Val638 and Glu673) has as yet been identiffied. To determine whether this region was protected from genetic variation we researched various databases and highlighted three rare variants: Cys633Ser (rs1126555), Asp636Asn (rs749873100), and Glu673Lys (rs368974006) (Ensembl database). Modeling of these residues showed that Cys633Ser and Glu673Lys indeed do have a deep structural impact: the first by disrupting a disulfide bridge, and the second by pushing away the Ca2+ atom by steric encumbrance. The presence of these potentially damaging mutations presumed to exist as very rare single alleles in a control population is compatible with the fact that heterozygotes for GT generally do not bleed (George et al., 1990). It would also be consistent with a constantly changing *ITGA2B* and *ITGB3* mutation repertoire in the general population (Buitrago et al., 2015; Nurden & Pillois, 2017).

Fundamental to our understanding of the structural effects of genetic variants affecting the αIIb genu is the conformation of the integrin during early αIIbβ3 biosynthesis and much less is known here. Of the 16 disease‐contributing variants that form the basis of our study, all but two give rise to type I GT consistent with a severe block in platelet αIIbβ3 expression; only Ala477Pro and Cys705Arg give rise to type II disease and low amounts of αIIbβ3 on platelets. Crucially, it has been suggested that proαIIbβ3 rapidly adopts a bent conformation during biogenesis (Mitchell, Li, French, & Coller, 2006; Mitchell et al., 2007). Evidence for this was obtained through the use of monoclonal antibodies specific for conformational‐dependent epitopes on αIIb or β3. Pro‐αIIb itself acquires a bent conformation soon after synthesis possibly through the interaction of the β‐propeller with calnexin or another membrane‐bound chaperone (Mitchell et al., 2006). β3 then adopts a bent conformation on binding to pro‐αIIb also in a chaperone‐dependent process. Pro‐αIIbβ3 complex formation occurs in the ER of MKs or transfected heterologous cells and following carbohydrate processing the complexes are transported to the Golgi apparatus where maturation including cleavage of pro‐αIIb into heavy and light changes occurs prior to passage of mature αIIbβ3 to the membrane systems of platelets. Early adaptation of the bent conformation during αIIbβ3 biosynthesis is highly compatible with the fact that pro‐αIIbβ3 formation was seen when αIIb477Pro and αIIb705Arg were expressed in β3‐containing heterologous cells (Fu et al., 2005; Gonzalez‐Manchon et al., 1999). However, the mechanisms by which the structural modifications of the thigh, genu and calf‐1 as we have observed for missense mutations by in silico modeling cause the block in αIIbβ3 maturation will require further study. Among the hypotheses that can be put forward is a reduced ability to bind chaperones necessary for pro‐αIIbβ3 transport from the ER to the Golgi apparatus. Not least, the structural destabilization appears to cause retention of the variant integrin in the ER with subsequent degradation by the megakaryocyte proteosome. While our results validate the use of in silico modeling as a rapid and inexpensive way of confirming the probable pathogenicity of GT‐causing missense mutations, they also urge caution in using this approach alone, with additional long‐range molecular dynamic simulations and site‐directed mutagenesis necessary for a complete armamentarium.

## DISCLOSURES

None of the authors have any conflicts of interest to disclose with respect to the work described in this paper.
