# Supplementary figures and images for: In silico analysis of structural modifications in and around the integrin αIIb genu caused by ITGA2B variants in human platelets with emphasis on Glanzmann thrombasthenia
Source: Mol Genet Genomic Med. 2018 Jan 31;6(2):249–60. doi: 10.1002/mgg3.365 (PMC5902390; doi:10.1002/mgg3.365)

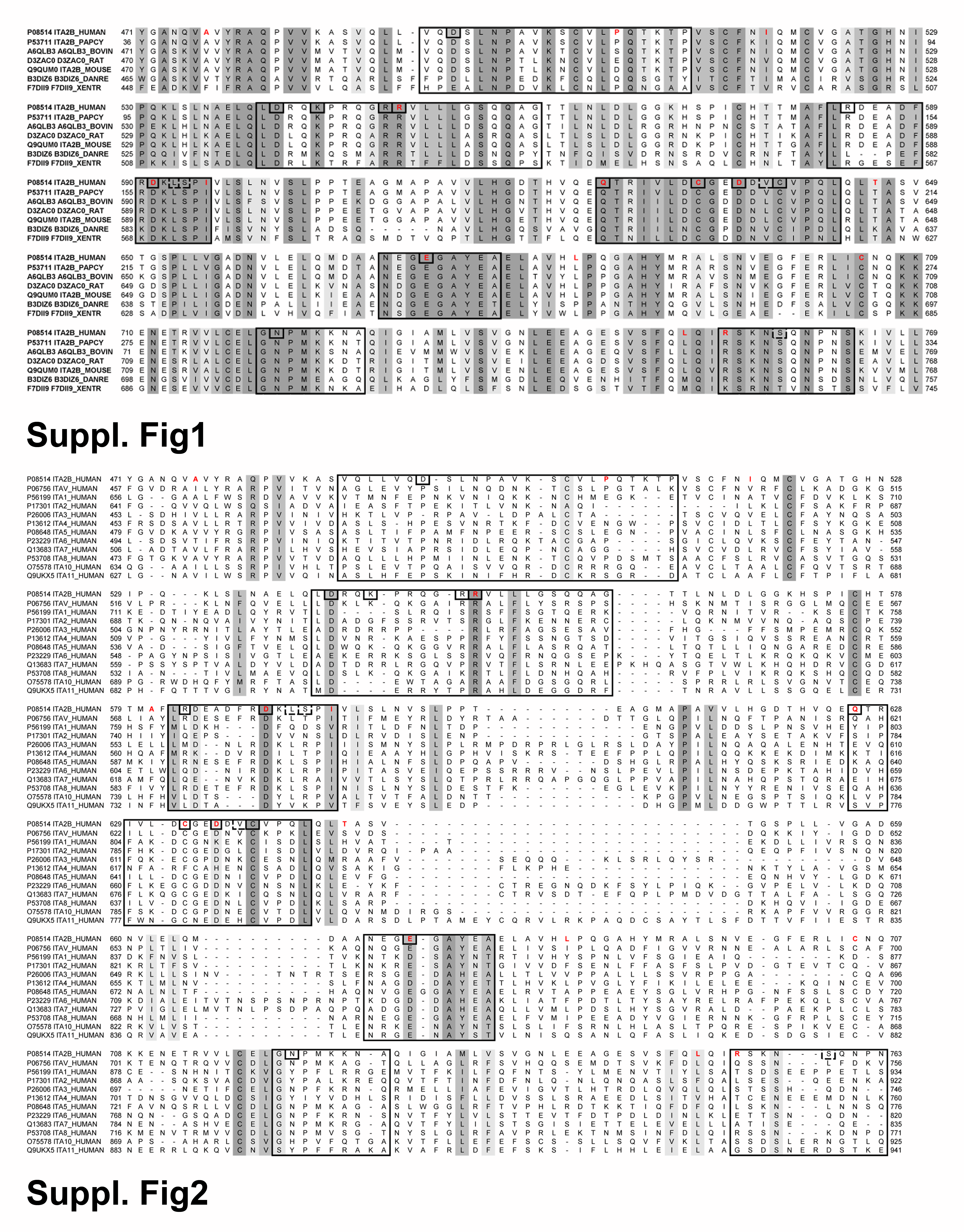

Supplement: Supplementary file 1 [file MGG3-6-249-s001.tiff]

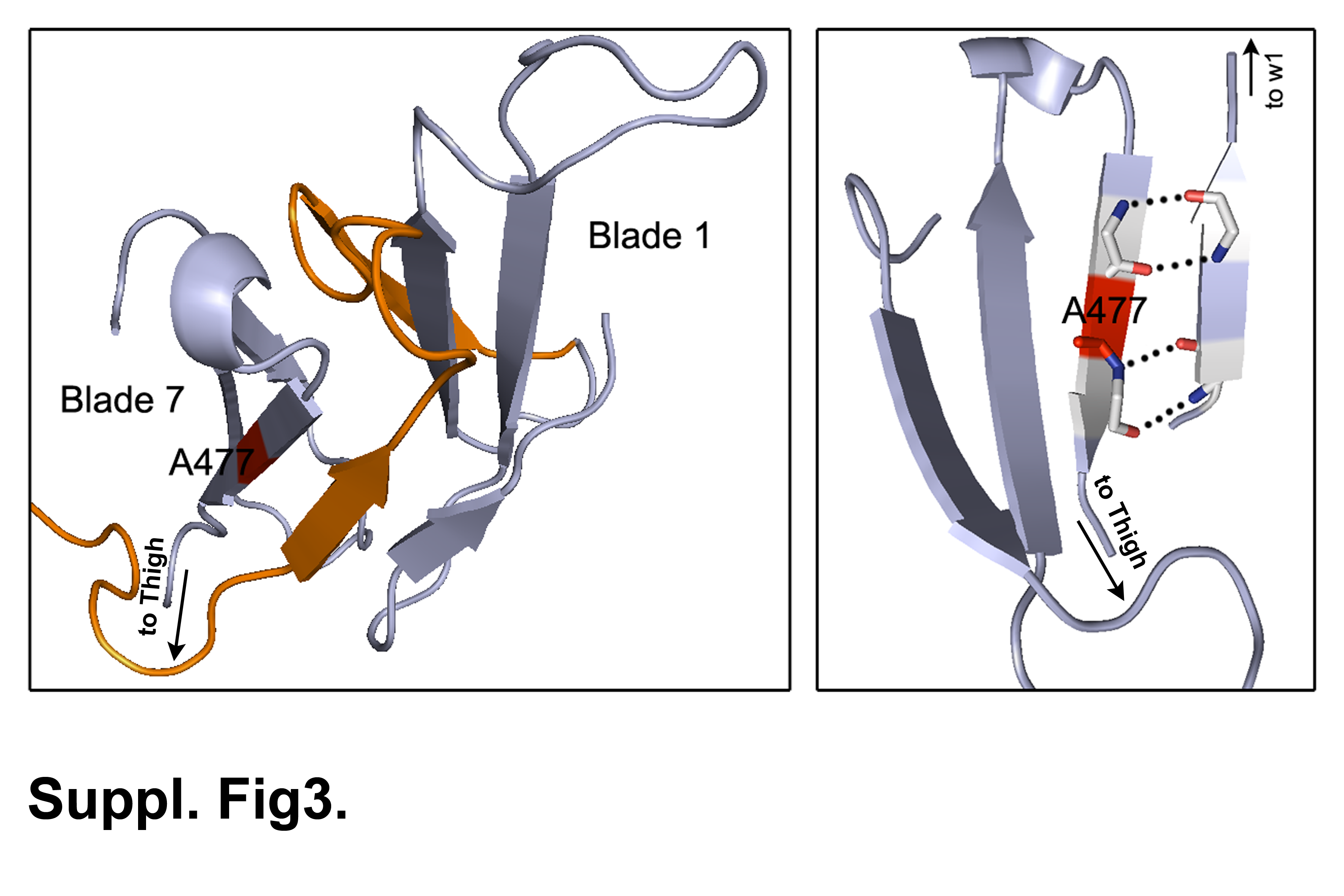

Supplement: Supplementary file 2 [file MGG3-6-249-s002.tiff]

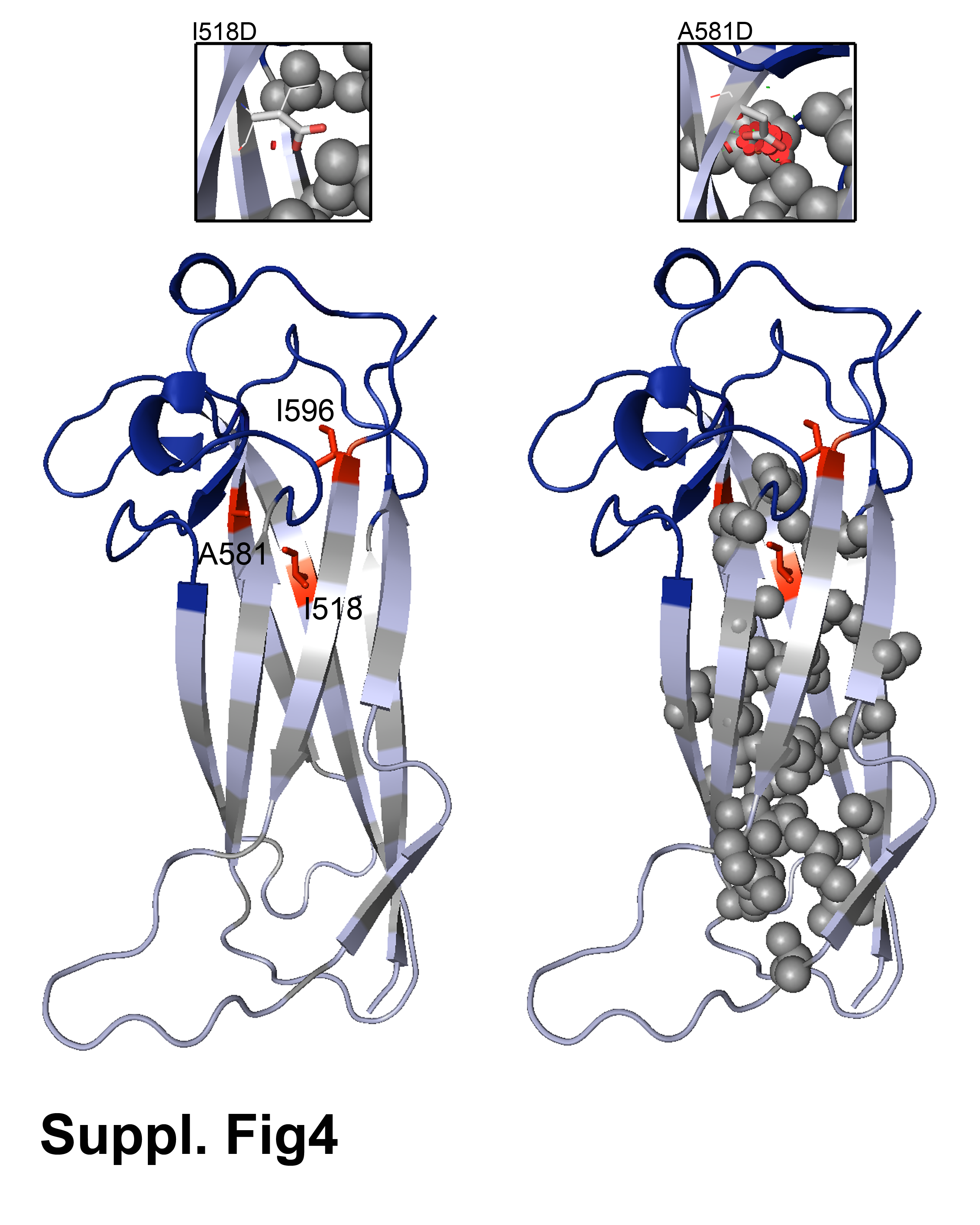

Supplement: Supplementary file 3 [file MGG3-6-249-s003.tiff]
