# Supplementary material for: In silico analysis of structural modifications in and around the integrin αIIb genu caused by ITGA2B variants in human platelets with emphasis on Glanzmann thrombasthenia
Source: Mol Genet Genomic Med. 2018 Jan 31;6(2):249–60. doi: 10.1002/mgg3.365 (PMC5902390; doi:10.1002/mgg3.365)
Supplement: Supplementary file 4 [file MGG3-6-249-s004.docx]

**Supp. Table S1: Clinical and biological data for previously reported GT cases with misssense mutations occurring in the aa471-745 region of αIIb**

| **Case** | **Gender** | **Origin^1^** | **Platelet aggregation** | **αIIbβ3 expression^2^** | ***ITGA2B* Missense variant** | **αIIb domain** | **Bleeding symptoms** | **Other comments** | **Reference** |
| --- | --- | --- | --- | --- | --- | --- | --- | --- | --- |
| 1 | M | China | Absent | 14% | Ala477Pro | **β**-propeller | Recurrent epistaxis, gum bleeding and purpura from birth | Expression studies in CHO cells | Fu et al., 2005 |
| 2 | F | India | Absent | NA | Pro507Arg | thigh | NA | Individual clinical data not given (cohort of 40 families) | Peretz et al., 2006 |
| 3 | M | USA | Absent | >5% | Ile518Asn | thigh | Severe life-threatening bleeding | Compound heterozygous with a splicing mutation (c.1946+3G>T) | Nurden et al., 2015 |
| 4,5 | M | India | Absent | >5%, 8% | Arg551Trp | thigh | Bleeding from multiple sites | None | Nelson et al, 2006 |
| 6-11 | M, F | India, Pak | Absent | <5% | Arg551Gln | thigh | Moderate to severe bleeding | Two independent studies with multiple families in Pak | Vijapurkar et al., 2009; Haghighi et al., 2016 |
| 12 | M | Italy | Absent | <5% | Ala581Asp | thigh | Moderate bleeding | Presumed compound heterozygous with a econd unknown mutation | D'Andrea et al., 2002 |
| 13-18 | M, F | NA, Sw, Fr, Ger | Absent | 5%/<5% when homozygous | Ile596Thr | thigh | Moderate to severe bleeding when data available | Compond heterozygous or homozygous | French & Coller, 1997; Ruan et al., 1998; Jallu et al., 2010; Sandrock et al., 2012; Sandrock-Lang et al., 2015 |
| 19-23 | M, F | Fr/N Afr, Italy, Ger | Absent | <5% | Gln626His | abnormal splicing | Moderate to severe bleeding when data available | Mutation known to give rise to abnormal splicing and mRNA decay. Expression in COS-7 cells | Pilliteri et al., 2010; Jallu et al., 2010; Sandrock et al., 2012; Nurden et al., 2015 |
| 24 | NA | Turkey | Absent | <5% | Thr646Ala* | calf-1 | NA | Individual clinical data not given | Tokgoz et al., 2015 |
| 25 | F | Italy | Absent | <5% | Leu684Arg | calf-1 | Severe bleeding | Compound heterozygous | Pillitteri et al., 2010 |

| 26-33 | F, M | Fr/Sp/Italy | Absent | 8-20% | Cys705Arg | calf-1 | NA/mild to severe bleeding | Compound heterozygous or homozygous. Expression in CHO cells | Gonzalez-Manchon et al., 1999; D'Andrea et al., 2002; Mitchell et al., 2003; Jallu et al., 2010; Nurden et al., 2015 |
| --- | --- | --- | --- | --- | --- | --- | --- | --- | --- |

| 34 | F | Ger | Absent | <5% | Cys705Leu | calf-1 | Moderate to severe bleeding that prompted transfusion | Identified mutation was heterzoygous; presumed 2^nd^ non-identified mutation | Santoro et al., 2010 |
| --- | --- | --- | --- | --- | --- | --- | --- | --- | --- |
| 35,36 | F | USA | Absent | <5% | Arg755Gln | calf-1 | Severe bleeding | Severe bleeding, Bone marrow transplant | Nurden et al., 2015 |
| 37 | F | India | Absent | <5% | Arg755Pro | calf-1 | Mild bleeding | Loss of pregnancy | Vijapurkar et al., 2009 |
| 38,39 | F | Italy | Absent | <5% | Leu752Val + Arg755Pro | calf-1 and calf-1 | Moderate bleeding | Compound homozygous expression | D'Andrea et al., 2002 |

^1^ Country of residence/origin; ^2^Surface expression determined by flow cytometry (; homozygous mutations are underlined. *Homozygosity suspected but not confirmed; NA, data not available; M, male; F, female; Pak, Pakistan; Sw, Switzerland ; Fr, France; Sp, Spain ; N Afr, North African; Ger, Germany;
