# Supplementary material for: In silico analysis of structural modifications in and around the integrin αIIb genu caused by ITGA2B variants in human platelets with emphasis on Glanzmann thrombasthenia
Source: Mol Genet Genomic Med. 2018 Jan 31;6(2):249–60. doi: 10.1002/mgg3.365 (PMC5902390; doi:10.1002/mgg3.365)
Supplement: Supplementary file 5 [file MGG3-6-249-s005.docx]

**Legends for Supplementary Figures**

**Supp. Figure S1 and S2.** Peptide sequence alignment of αIIb (region from aa471 to aa769) through species (orthologue; Fig. 1 top), and through different members of human integrin α-subunits family (paralogue; Fig. 2 bottom). The intensity of the gray is a measure of amino acid conservation or deviation. Framed are amino acids constituting the genu – note that they are not continuous. Plain boxes highlight amino acids participating in structural H-bonds through their side chain, while dashed boxes highlights amino acids participating in structural H-bonds through their main chain within the genu.

**Supp. Figure S3.** Cartoon diagram of Blade 7 and Blade 1 of the αIIb propeller. In the left window, amino acids sequence linking blade 7 to blade 1 and closing the propeller structure are colored in orange. In the right window, H-bonds linking β-strands 3 and 4 of Blade 7 are highlighted. Alanine 477 is colored in red. Models were obtained using the PyMol Molecular Graphics System, version 1.3 and the 3fcs pdb file.

**Supp. Figure S4.** Ribbon diagrams highlighting missense mutations Ile518Asp and Ala581Asp (red sticks) within the calf-1 domain of αIIb. Hydrophobic amino acids whose side chain point to the core of the barrel are in gray and represented as spheres in the right representation. In small windows are enlarged views with each mutation illustrated as graphical sticks with superimposed, the natural amino acid represented as lines. C atoms are colored in white, N atoms in blue, O atoms in red and S atoms in orange. Graphical “bumps” (red discs) reveal steric encumbrance caused by the amino acid substitution. Models were obtained using the PyMol Molecular Graphics System, version 1.3 and the 1uv9 pdb file.
